# Molecular Aspects of Sex Development in Mammals: New Insight for Practice

**DOI:** 10.3390/ijms21239146

**Published:** 2020-11-30

**Authors:** Laura Audí, Silvano Bertelloni, Christa E. Flück

**Affiliations:** 1 Growth and Development Research Group, Vall d’Hebron Research Institute (VHIR), Center for Biomedical Research on Rare Diseases (CIBERER), Instituto de Salud Carlos III, Barcelona, 08035 Catalonia, Spain; 6620lap@gmail.com; 2 Pediatric and Adolescent Endocrinology, Division of Paediatrics, Azienda Ospedaliero-Universitaria Pisana, 56126 Pisa, Italy; 3 Pediatric Endocrinology, Diabetology and Metabolism, Department of Pediatrics and Department of BioMedical Research, Bern University Hospital and University of Bern, CH-3010 Bern, Switzerland; christa.flueck@dbmr.unibe.ch

Disorders (or differences) of sex development (DSD) are congenital conditions characterized by atypical development of genetic, gonadal or phenotypic sex. This terminology recognizes the simple but fundamental biology of the nature of sex chromosome (XY or XX) organizing the development of the gonads (testis or ovary) whose hormones (effectively androgens in fetal life) then determine the typical genital phenotype (male or female) and likely cause an imprinting of the fetal brain [1].

DSDs include a wide spectrum of conditions mostly due to genetic variants, altered hormonal secretion or abnormal peripheral sensitivity to gonadal hormones, that are all able to change the typical male or female fetal development. The impact of DSD on the quality of life of affected persons and their families is remarkable, as most DSD conditions involve complex clinical, endocrinological and psychological challenges. Expert opinion and shared decision are required, e.g., for sex assignment at birth (and eventually re-assignment later on), evaluation about gonadal surgery, because of the risk of gonadal neoplasia, hormone replacement therapy from adolescence onward, fertility counselling, and holistic long-term monitoring and preventive care of health. Thus, optimal management of individuals with DSD requires expert knowledge in this very specialized area, which is available in interdisciplinary DSD teams working at centers of excellence for DSD care [2,3].

In the last two decades, impressive improvements in both clinical as well as research approaches have been reached, allowing better understanding of genetic and physio-pathologic basis of old and new DSDs. National as well as international networks have been established and an international registry, namely the I-DSD (https://home.i-dsd.org/) has been recognized as common database, increasing the number of DSD individuals contributing with their data towards better knowledge on DSDs and their long-term outcome. The future challenge for health professionals will lie in integrating specific basic information with comprehensively defined clinical and endocrine phenotypes as well as long-term consequences. Such advances will permit to optimize the care of patients and the counselling for parents. Concerning the perspective of societies on DSDs, several countries and cultures significantly enhanced their sensitivity on the topic and changed their opinion/attitude to accept and include persons with a DSD and expand the strict binary male and female sex/gender categories with either a third, atypical or gender-neutral category [2,4]. 

This themed issue on DSD of the International Journal of Molecular Sciences presents a series of articles written by colleagues who have been recognized as true experts in each chosen area of DSD. They cover several hot topics, provide some state of the art papers on specific areas and update some research or management aspects. 

Ali et al. [5] summarize the long experience of the International Disorders of Sex Development (I-DSD) and International Congenital Adrenal Hyperplasia (I-CAH) registries. They demonstrate that the registries are indispensable tools for monitoring clinical and patient-centered outcomes. The authors also demonstrate that registries allow to gather large enough cohorts in DSDs by strict collaborative efforts to perform meaningful research on rare conditions.

Neirijnck and co-workers [6] address a poorly explored issue that is the roles of insulin/IGF system in sexual development and reproduction of both males and females. They highlight some interesting new findings and unanswered questions that remain open for additional studies.

On the basis of their long-lasting clinical and laboratory experience, Baronio et al. [7] update genetic, biochemical, and clinical features of 46, XX DSD due to androgen excess in monogenic disorders of steroidogenesis. They elaborate on the precise role of steroidogenic enzymes and cofactors, stressing that understanding steroidogenesis is a key factor for the comprehension of DSDs and other processes related to human reproduction and fertility.

The risk of gonadal germ cell cancer (GGCC) is increased in some patients with DSD [2]. Looijenga and colleagues [8] discuss the possibility to stratify individuals with an increased tumor risk with parameters other than histological investigation of a gonadal biopsy. They summarize current opportunities as well as limitations in predicting the personal risk of GGCC in a single specific individual with DSD.

Rodríguez Gutiérrez et al. [9] explore the revolutionary techniques of cellular reprogramming and guided in vitro differentiation, permitting to exploit the versatility of induced pluripotent stem cells to create alternative models for DSD, ideally on a patient-specific personalized basis.

Grinspon and Rey [10] provide insight into the molecular explanation of XX maleness. It frequently occurs in XX individuals in the presence of a translocated *SRY* on a X chromosome or autosome. However, new genomic technologies allow the discovery of novel mechanisms explaining the development of testicular tissue in the absence of *SRY* expression in the embryonic undifferentiated gonads.

Camats et al. [11] explain that the broad phenotypes of individuals with DSD may be due to oligogenic inheritance instead of single gene defect. Based on their own research in carriers of *NR5A1* variants and of *MAMLD1* variants, they demonstrate that multiple genetic hits may contribute to a specific DSD phenotype, unique to each individual. This also suggest that multiple genes work together in a concerted network for typical sex development.

Tyutyusheva and colleagues [12] report on the molecular basis of complete androgen insensitivity syndrome and discuss some practical topics including management of the gonads, hormonal replacement therapy during adolescence and adulthood, bone health, and metabolism.

Ristori et al. [13] raise a very hot topic. They report on the impact of sex hormones and genetic background on brain sexual differentiation and gender identity, concluding that the sexual dimorphic brain could be the anatomical substrate of dimorphic psychosexual development, on which gonadal hormones may have a shaping role during prenatal and pubertal periods. 

The authors contributing to this Special Issue of the Journal have been selected based on their high-quality expertise and their abilities to make complex topics available to all health care providers. We hope that the readers will find this themed issue not only enjoyable but also helpful for the care of individuals with DSD.

We would like to take the opportunity to express our gratitude and thanks to all the authors involved in this selected issue for their high-level papers. We also thank the reviewers for their time and expertise, as well as the Chief Editors of the International Journal of Molecular Sciences for giving us the honor of publishing this special issue.
